# The Creation of Artificial Data for Training a Neural Network Using the Example of a Conveyor Production Line for Flooring

**DOI:** 10.3390/jimaging11050168

**Published:** 2025-05-20

**Authors:** Alexey Zaripov, Roman Kulshin, Anatoly Sidorov

**Affiliations:** Department of Data Processing Automation, Tomsk State University of Control Systems and Radioelectronics, 634050 Tomsk, Russia; aleksei.v.zaripov@tusur.ru (A.Z.); roman.s.kulshin@tusur.ru (R.K.)

**Keywords:** data generation, neural network, synthetic data, computer vision, YOLO, Unity, conveyor, laminate, defect

## Abstract

This work is dedicated to the development of a system for generating artificial data for training neural networks used within a conveyor-based technology framework. It presents an overview of the application areas of computer vision (CV) and establishes that traditional methods of data collection and annotation—such as video recording and manual image labeling—are associated with high time and financial costs, which limits their efficiency. In this context, synthetic data represents an alternative capable of significantly reducing the time and financial expenses involved in forming training datasets. Modern methods for generating synthetic images using various tools—from game engines to generative neural networks—are reviewed. As a tool-platform solution, the concept of digital twins for simulating technological processes was considered, within which synthetic data is utilized. Based on the review findings, a generalized model for synthetic data generation was proposed and tested on the example of quality control for floor coverings on a conveyor line. The developed system provided the generation of photorealistic and diverse images suitable for training neural network models. A comparative analysis showed that the YOLOv8 model trained on synthetic data significantly outperformed the model trained on real images: the mAP50 metric reached 0.95 versus 0.36, respectively. This result demonstrates the high adequacy of the model built on the synthetic dataset and highlights the potential of using synthetic data to improve the quality of computer vision models when access to real data is limited.

## 1. Introduction

In the implementation of a wide range of industrial processes based on multi-stage transformations of raw materials into finished products, there arises a need for quality control of intermediate and/or final results. This need is expressed through the identification and rejection of objects that do not meet specified parameters indicating compliance with certain standards, regulations, or norms. Typically, this task is performed through human visual inspection, which involves several issues:

- low speed of evaluation and classification (compliant/non-compliant with quality parameters) due to the physiological limitations of the human visual system;
- subjectivity of perception, which may lead to missed detection of substandard objects or incorrect classification caused by factors such as the quality inspector’s qualifications, fatigue, loss of concentration, etc.;
- costliness of this stage in the production process, which does not directly contribute to the added value of the product and includes, among other things, the need to compensate specialized personnel.

To address these challenges, various digital solutions based on computer vision (CV) systems are increasingly being adopted. These systems are being integrated across multiple industries and specific production tasks, including: traffic monitoring [1,2,3,4], metallurgy [5,6,7,8], perimeter security and safety compliance [9,10,11,12], agriculture [13,14,15], and others. The technology enables automation of numerous routine and attention-intensive tasks.

For example, in [16], potential applications of CV technology are discussed in the context of detecting and classifying diseases in various medical images, such as ultrasound scans and microscope images of tissue samples.

Computer vision can also be applied in the printing industry to enhance the effectiveness of quality control over printing equipment, ultimately reducing the cost of the final product. During the technological process, components such as engraved printing cylinders are produced, which are used to apply images or text onto paper. In [17], deep neural networks were trained to detect defects (dents, scratches, inclusions, bends, misalignments, excessive, faded or missing prints, and color errors resulting from engraving). The training data consisted of photographs of printing machine components, classified by operators as either defective or defect-free. During testing, the model achieved an accuracy of 97.85% for true negative results, and 99.01% for true positives—clearly indicating a high level of performance for the given task.

Building effective computer vision (CV) systems requires a preliminary data collection stage, which includes obtaining images of relevant objects along with their coordinates within the image. However, in many fields, acquiring a diverse and timely dataset is challenging, as technological and other processes may not allow for the reproduction of rare, hazardous, or economically inefficient scenarios. Additionally, manual image annotation is subject to human error, which can negatively affect the quality of neural network training.

Due to these limitations, obtaining uniform datasets becomes problematic, which reduces the generalization ability of neural networks and, consequently, their accuracy. One of the solutions to the data scarcity issue is the generation of artificial images using various methods. Currently, there are numerous approaches and implementations of data generators tailored to different subject domains.

## 2. The Concept of Data Generation

The problem of automating the data collection process is not new, and numerous studies have been conducted to address it. For example, the study [18] presents a review of image generation methods aimed at producing data for machine-learning systems, focusing on photorealistic rendering techniques such as path and ray tracing.

In article [19], various synthetic data generation methods for neural networks are described, discussing different types of generators—such as generative neural networks and simulators—that can be used to create images.

Some researchers [20] describe the process of generating artificial data by simulating UAV (unmanned aerial vehicle) behavior for detection and classification tasks using the video game GTA V (Grand Theft Auto V). However, current video games offer limited capabilities for data generation and do not support a wide range of diverse scenarios. To implement more atypical use cases not supported by video games, other software tools are used, including 3D modeling software such as Blender [21], ZBrush [22], and 3Ds Max [23].

For instance, Blender was used to build a synthetic data generator for detecting pipeline defects using UAVs [24]. The system provides a flexible dataset of photorealistic images and allows for customizable usage scenarios.

The article [25] proposes a method for generating synthetic images of defects, specifically chips, for use in various industrial applications. As an example, the study uses a turbocharger component. The proposed generator relies on procedurally generated textures applied to the object of interest to increase dataset diversity. Defects are simulated by cutting out randomly shaped segments from the 3D mesh, allowing for a wide range of potential damages. Test results showed that a model trained solely on synthetic data outperformed a model trained only on real images of turbocharger chips.

However, the Blender-based approach has limitations—it cannot produce a large number of frames per second. Even with a decent rendering speed of 1 fps, generating a one-minute video would take about 30 min. For faster data generation with only a slight reduction in image quality, game engines like Unreal Engine, Unity, and CryEngine are often used.

Unity, for example, includes the Unity Perception package, which offers tools for automated annotation of synthetic data in its native SOLO format. In [26], Unity and Unity Perception were used to generate training data for a neural network tasked with detecting manufactured metal bolts on a conveyor belt. This solution enabled data generation at up to 6 fps—significantly faster than Blender—but still did not fully leverage Unity’s potential.

The authors of [27] conducted an experiment to generate industrial safety data. A construction site with scaffolding was simulated using a game engine, and human behavior was animated using skeletal animation tools. Neural network models trained on synthetic data mixed with real samples achieved the highest performance.

The study [28] proposed a solution for generating data for traffic sign detection. Unity Perception was used to render 2D images from 3D models, separating them into detection objects, noise elements, and background layers. Although models trained only on synthetic data performed worse than those trained on real images, the time saved on data annotation was considerable. Moreover, a model trained on a mixed dataset outperformed both others, highlighting the value of synthetic data in machine learning.

CAD (computer-aided design) systems can also be used among the tools for working with 3D graphics, as, for example, in article [18]. Here, various 3D parts with diverse textures, viewpoints, and lighting conditions were used to produce a highly variable training set. Experiments demonstrated that neural networks trained on synthetic data were able to recognize additive components with high accuracy.

The authors of [29] consider a technique for generating synthetic images using CGI (computer-generated imagery) graphics based on thin shell simulation. This approach accurately replicates the photorealistic behavior of textile materials, including wrinkles and folds, which is important for tasks related to clothing structure analysis or other soft surfaces. Experimental results showed an 8–10% increase in prediction accuracy for models trained with synthetic data compared to those trained exclusively on real images—demonstrating the utility of CGI-based synthetic data in enriching computer vision datasets.

Another notable advancement is in the use of synthetic composite images (SCI)—real photographs digitally manipulated or augmented with elements not originally present. For example, the Super real dataset presented in paper [30] includes around 6000 Skins created for segmentation tasks. In this dataset, 3D models of people in various poses are overlaid onto real-world backgrounds. Experiments showed that neural networks trained on SCIs achieved higher segmentation accuracy compared to models trained solely on real images. The best performance was achieved by a model with an upsampling layer that increased image resolution before segmentation. This model was trained on a mixed dataset, combining both real and synthetic images, resulting in improved segmentation quality.

Study [31] also investigates composite image generation, where CAD models with various viewpoints and lighting conditions are superimposed on background images to increase dataset variability. While models trained solely on synthetic images generally underperformed compared to those trained on real data, freezing the feature extractor significantly improved their performance on real test data.

Neural networks themselves are also used to generate synthetic data. One such method involves Variational Autoencoders (VAEs), which include two key components: an encoder and a decoder. VAEs generate new images by learning the underlying data distribution, encoding input images into latent space and decoding them to generate new samples. In [32], a VAE was used to generate random faces. However, the resulting images were often blurry, particularly at high resolutions.

Another technique uses generative adversarial networks (GANs), consisting of a generator and a discriminator that compete against each other. The generator creates synthetic images, while the discriminator evaluates their authenticity. This adversarial process continues until the generator produces high-quality, realistic images [33].

In paper [34], a GAN-based method is proposed for training a neural network to detect and classify people. A “synthesizer” network generates images, which are then evaluated by a “target” network. A discriminator, trained on real images, helps the synthesizer avoid obvious artifacts and improve image realism.

In study [35], GANs are used to expand a dataset aimed at image segmentation. The generated images train a semantic segmentation network responsible for identifying defects. The method improves the model’s robustness to different defect types and lighting conditions, making it more effective in real industrial environments. The synthetic data’s diversity reduced overfitting to limited real-world datasets.

Each approach addresses the data shortage challenge in its own way, with its own strengths and limitations. For instance, 3D editor-based generation is relatively slow due to low rendering speed. The GTA V-based method does not support data generation for industrial processes, which is a significant drawback, but it allows real-time UAV simulation. Neural network-based methods such as VAE and GAN also have limitations—they require real data to train the models before generation can begin.

## 3. The Concept of Digital Twins

A digital twin is a dynamic virtual copy of a real object, process, system, or environment. It replicates not only the appearance but also the key properties of its physical counterpart, enabling detailed analysis, simulation, and forecasting of its behavior. The use of digital twins improves process efficiency, reduces maintenance costs, predicts potential failures, and enhances product quality [36].

In paper [37], the authors presented a classification of digital twins based on the level of integration:

- digital model: a static 3D representation without any connection to the real object;
- digital shadow: a model that is updated based on incoming data, but the connection is one-way—from the physical object to the model;
- digital twin: provides a two-way connection with the physical object, allowing not only data acquisition but also real-time simulation of changes.

Digital twins are used to solve various tasks related to testing and predicting the behavior of real objects.

For example, in [38], digital twins are discussed in the context of optimizing construction processes, reducing costs, and increasing efficiency through the integration of building information modeling (BIM) and IoT technologies.

In [39], the prospects of Industry 4.0—combining IoT and digital twins—are examined in the context of education. The study concludes that it is necessary to implement digital twin research programs, both conceptually and methodologically, with practical application in mind.

Study [40] explores the concept of digital twins in agriculture. Based on the analysis of existing definitions, a typology of digital twins was proposed according to lifecycle stages, including the following categories:

- Imaginary digital twin: a conceptual digital model representing an object that does not yet exist physically;
- Monitoring digital twin: a digital representation of the current state, dynamics, and trajectory of a real physical object;
- Predictive digital twin: a digital projection of possible future states and behaviors of physical objects, based on predictive analytics, including statistical forecasting, simulation, and machine learning;
- Prescriptive digital twin: an intelligent digital model capable of recommending corrective and preventive measures to optimize the operation of real-world objects. These recommendations are usually based on optimization algorithms and expert heuristics;
- Autonomous digital twin: a digital twin with autonomous functions, capable of fully controlling the behavior of physical objects without human intervention, either locally or remotely;
- Recollection digital twin: a digital representation containing the complete history of a physical object that no longer exists in reality.

Additionally, a hardware–software solution was developed in the study to implement and experimentally test the concept of digital twins in agriculture.

Study [41] provides a comprehensive review of the application of digital twin technology in the context of intelligent electric vehicles. The research focuses on optimizing electric transport operations through the use of digital twins for real-time monitoring and prediction of the state of key vehicle components and systems. Digital twin technology improves the efficiency of electric vehicle operation by enabling better use of energy and material resources. In particular, timely identification of potential failures and optimization of operational parameters not only extends component lifespan, but also significantly reduces the environmental impact of transport.

In paper [42], the digital twin concept is used to optimize pig farming in agriculture. A “pig twin” model was developed to track growth under varying conditions and optimize feeding. The pig digital twin is currently under development and will be used to explore the potential of a closed-loop control system at the Industry 4.0 level.

Unlike agriculture, which focuses on adaptation to natural conditions and biological factors, industry demands higher precision, reliability, and integration of digital solutions into complex production chains. In this context, digital twins are a key tool in digital transformation, enabling continuous process optimization, predictive maintenance, and cost reduction.

For creating digital twins of conveyor-based production, game engines are often used. For instance, the company Prespective developed software integrated with the Unity game engine, allowing rapid design of conveyor lines. Using this solution, an automotive assembly process was recreated, enabling assessment of line performance under equipment failure conditions for incident prioritization. Moreover, the system provides operators with detailed order information necessary for proper vehicle assembly [43].

Study [44] proposes a fault diagnosis methodology for permanent magnet synchronous motors used in coal conveyors, based on digital twin technology. The proposed approach integrates the digital twin concept with an optimized random forest algorithm. A bidirectional data transmission system was implemented to synchronize the physical object and its virtual copy in real time. Simulation results, confirmed by experimental data, showed a diagnostic accuracy of 98.2%, which indicates that 98.2% of failures were correctly predicted. This study highlights the potential of digital twins for motor condition monitoring and emphasizes further integration of fault diagnosis with 3D visual control of equipment operation.

Study [45] presents a comparative analysis of robotic arm digital twin development using two software platforms: Unity and Gazebo. The study evaluated the performance of these environments in creating dynamic digital models. The development was based on a unified physical setup and communication layer, ensuring the objectivity and accuracy of the comparison.

The results showed that Unity has advantages in simulation accuracy and lower response latency, making it optimal for applications requiring high visualization precision and fast data processing. On the other hand, Gazebo offers faster integration with the Robot Operating System (ROS), making it preferable for low-budget robotics and automation projects.

Digital twins are also suitable for generating synthetic data, as they allow simulation of system operation under extreme conditions without the need to reproduce dangerous scenarios.

Study [46] is dedicated to the digital twin of a wind turbine energy conversion system. A hybrid model was developed to generate synthetic data for fault diagnosis.

In the context of generating synthetic images, basic-level digital twins—digital models—are sufficient, since a real-world connection is not necessary to obtain high-quality, realistic data for neural network training.

For instance, Boeing used a digital model of its aircraft for an AR (Augmented Reality) inspection application, generating over 100,000 synthetic images to train machine-learning algorithms [47].

To achieve the goal of synthetic data generation, a simple digital twin of the studied object must be created. This approach allows for the rapid development of training datasets using digital models that serve as a specific implementation of the digital twin concept.

## 4. Generalized Model of the Synthetic Data Generator

The task of identification involves detecting the object of interest that needs to be recognized and classified by the computer vision (CV) system. For example, in quality control of brick production, the object of interest would be brick defects. If the task is to detect vehicles using UAVs, the objects of interest would be various types of vehicles.

To address identification tasks within a conveyor-based manufacturing process, a generalized model for generator formation is proposed. This model consists of a set of interconnected tasks and simulates the production process:

- Creation of a digital twin (digital model) of the conveyor line (generation of 3D models of the conveyor belt; generation of 3D models of the conveyor sidewalls; texturing of the belt and sidewall models);
- Creation of the object of interest (generation of 3D models of the object of interest; texturing of the object models; development of algorithms for modifying the object of interest);
- Simulation of the real technological process (development of algorithms for the movement of objects of interest along the digital twin of the conveyor line; development of algorithms for modifying the digital twin and applying effects; development of algorithms for changing the production camera’s viewing angle).

The step-by-step implementation of the above-mentioned tasks enables the creation of a synthetic data generator framework suitable for any conveyor-based manufacturing process. As an example, a specific case of modeling laminate production will be considered further in this work.

**Figure 1:**
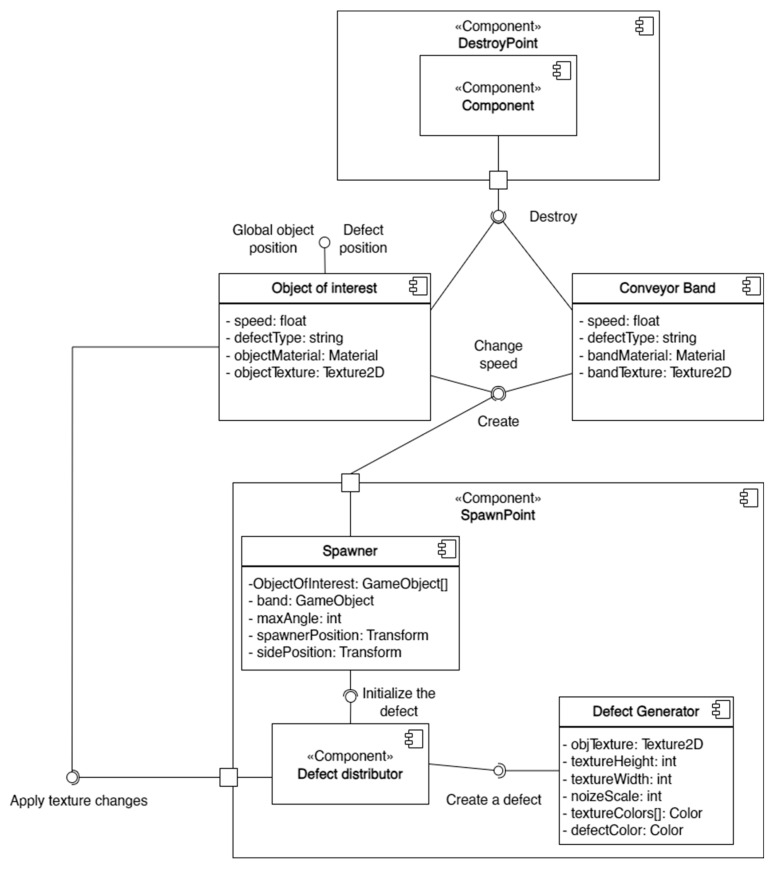
Diagram of software components.

Within the scope of the generalized task list, specialized software was developed, with its component diagram shown in Figure 1.

Scene generation was carried out through the operation of eight main components responsible for the creation and destruction of objects of interest, defect overlay, simulation of conveyor line operation, and expansion of the image dataset.

Situations are generated by the work of 8 main components of the scene responsible for the creation and destruction of objects of interest, the imposition of defects, the simulation of the conveyor line, and the expansion of the image sample.

### 4.1. Formation of the Digital Twin of the Conveyor Line

The system is implemented using the Unity game engine, which enables real-time scene rendering, along with its associated programming language, C#, and High-Definition Render Pipeline (HDRP) technology, which provides high-quality image generation.

**Figure 2:**
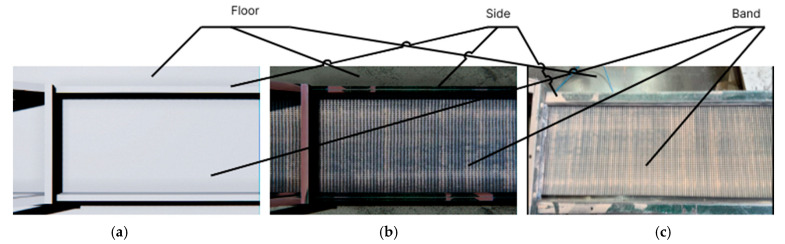
Conveyor line: (**a**) representation of primitives; (**b**) textured model; (**c**) real prototype.

Since the generalized task list involves the creation of a digital model, a representation of the conveyor was constructed using engine primitives (Figure 2a), specifically parallelepipeds. High-resolution textures were then applied to this representation (Figure 2b), which were created based on a real-life prototype—a conveyor line from a flooring production facility (Figure 2c).

The result of completing the first task from the generalized model was a generated 3D model of the conveyor, which was used to simulate the production process.

### 4.2. Formation of an Object of Interest

The object of interest in the given process is a laminate board, which must later be detected by the CV system. Accordingly, it is important to reflect the maximum number of object variations within the synthetic dataset. To create the objects of interest, ten high-resolution laminate board textures and their corresponding normal maps were developed. The number of textures was chosen to cover the main visual and textural variations commonly found in real-world conditions. This amount is sufficient to generate a large enough volume of synthetic data required for proper training of the CV system. The textures were subsequently applied to 3D parallelepiped objects.

Next, algorithms for modifying the boards were developed. To increase the diversity of the dataset, it was decided to expand the number of objects of interest by applying defects. For this purpose, two components were created: the Defect Distributor and the Defect Generator (Figure 1).

The Defect Distributor manages the defect application process. It performs the following tasks:

- Randomly determines whether a defect should be created;
- Modifies the object to allow proper defect application (adds annotation markers, creates additional empty objects);
- Randomly selects a defect from the available list;
- Transfers the prepared object to the defect application procedure.
- The Defect Generator is responsible for creating three types of defects:
- Print defect—an area of the printed pattern on the board that differs in color and texture from the main surface;
- Glue spot—a light gray spot on the outer surface of the laminate, caused by glue seeping from the adhesive layer beneath;
- Corner chipping—chips along the edges of the board, either across its entire length or in partial sections.

The defect selection algorithm is based on generating a random number from 0 to 1, which helps to distribute defects proportionally and maintain class balance in the training dataset.

**Figure 3:**
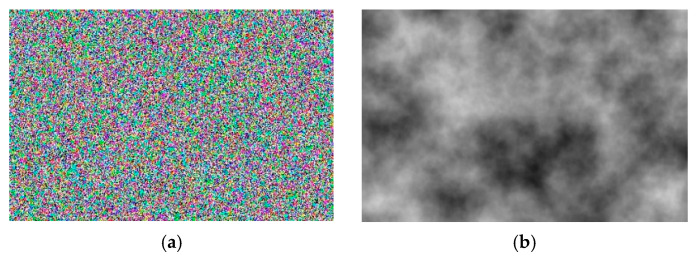
Noise visualization: (**a**) uniformly distributed noise; (**b**) Perlin noise.

The print defect and glue spot are generated using an algorithm based on Perlin noise, implemented with Unity’s built-in tools. Perlin noise is a type of gradient noise that uses random number generation. Unlike uniformly distributed noise (Figure 3a), where each new value can vary sharply from the previous one, Perlin noise (Figure 3b) ensures smooth transitions between values.

Using Perlin noise makes it possible to create a smooth random pattern texture, which enhances realism and is applied in various fields such as medicine [48], information security [49], and terrain generation [50]. Perlin noise was chosen as the basis for the spot generation algorithm because this type of procedural generation allows for the creation of smooth, pseudo-random textures on the surface of the object of interest.

Initially, a spot area is selected on the laminate board with a random size, which does not exceed the distance from the center of the stain to the nearest edge of the object. Once the spot area is defined, the noise value of each texture pixel is calculated within the range [0, 1]. Then, for values below a specified threshold, the pixel color is set to the predefined spot color.

**Figure 4:**
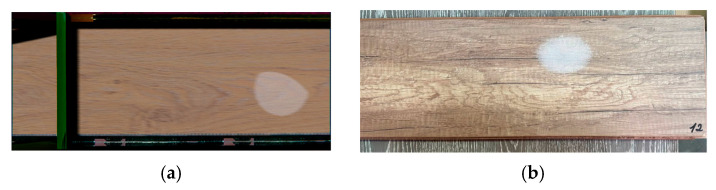
An example of the formation of a “glue spot” defect: (**a**) synthetic image; (**b**) real image.

**Figure 5:**
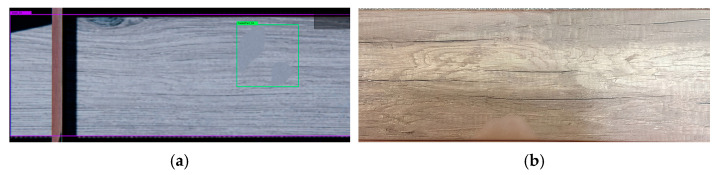
An example of the formation of a “print defect”: (**a**) synthetic image; (**b**)real image.

To prevent repetition of spots generated using noise, the seed value must be changed to a randomly assigned number. The results of the spot generation process are shown in Figure 4 and Figure 5. The proposed algorithm enables the generation of pseudo-random texture spots on the board surface. This significantly expands the sample of objects of interest and facilitates training of the neural network on a wider variety of data. As a result, the training quality improves, which positively affects subsequent object identification in manufacturing processes.

The algorithm responsible for corner chip generation is based on altering the transparency of a selected pixel—specifically, by reducing its alpha channel value from 100 to 0. Initially, the algorithm randomly determines the number of corner chips (from 1 to 4), corresponding to the number of board corners. Then, based on this number, corners to be “chipped” are randomly selected, and the defect creation process began.

**Figure 6:**
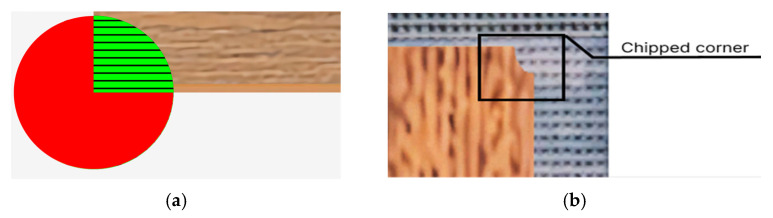
Demonstration of chip generation: (**a**) is the sector of the circle defining the chip; (**b**) is the result of chip formation.

Once a corner is selected for chipping, the chip size is randomly determined, represented as the radius of a circle centered at the extreme pixel of the corner. For example, if the board texture is sized 2048 × 512, the chip centers can be pixels at coordinates (0, 0), (0, 512), (2048, 0), and (2048, 512). The chip itself is a sector that extends into the board area from a circle positioned at the texture’s edge. In Figure 6a, a circle with a radius of 100 pixels is shown at a board corner with dimensions of 4096 × 980. The shaded area indicates the circle sector where pixel alpha channel values will be modified, while the unshaded area remains unaffected.

Next, the pixels within the chipped area must be altered accordingly. To do this—as with the algorithms for generating “glue spots” and “print defects”—the circle equation must be used.

If a pixel with coordinates *x* and *y* is located within a circle of *radius*, its alpha channel value is set to 0; otherwise, the pixel remains unchanged. The result is shown in Figure 6b. For clarity, the defect was highlighted with a rectangle with the inscription in the picture.

As a result of implementing the algorithms described above, the number of board variations increased significantly from an initial set of 10. Consequently, such a large dataset of diverse objects will allow the neural network to be treated without using identical images, which will positively impact its generalization ability and detection accuracy.

### 4.3. Simulation of a Real Technological Process

To simulate conveyor-based production, the components “object of interest”, “conveyor belt”, and “spawner” were created (see Figure 1). The “spawner” component is responsible for generating variations of the object of interest and randomly adding them to the scene along with the conveyor belt component. Before placing the object on the scene, its position is randomly determined based on the dimensions of the conveyor belt. The offset of the object of interest from the center of the conveyor belt is set within the range [−*distance*, *distance*], where the *distance* is calculated using the following formula:

(1) $$distance=\left|pos_{1}-pos_{2}\right|$$

where *pos*_1_ is the position of the “spawner” object; *pos*_2_ is the position of the “Side” object.

After the object’s offset is selected, its rotation angle in space is also randomly chosen from the range [−*max_angle*, *max_angle*] to prevent it from going out of bounds or colliding with other objects in the scene. The maximum rotation angle is calculated using the following formula:

(2) $$max\_angle=arctg\left(\frac{w}{l/2}\right)$$

where *w* is the distance from the object of interest to the nearest edge of the conveyor belt, and *l* is the length of the object of interest.

The “object of interest” and “conveyor belt” components are responsible for storing information about the object’s position and for moving it through space from the creation point to the destruction point at a specified speed, which is set during the generation setup phase in the parameters section.

To achieve greater image diversity, an algorithm was developed to alter the color of the conveyor line. This helped prevent the model from overfitting to similar images, which would reduce its generalization ability and significantly lower prediction accuracy on data that differs from the training set [51].

**Figure 7:**
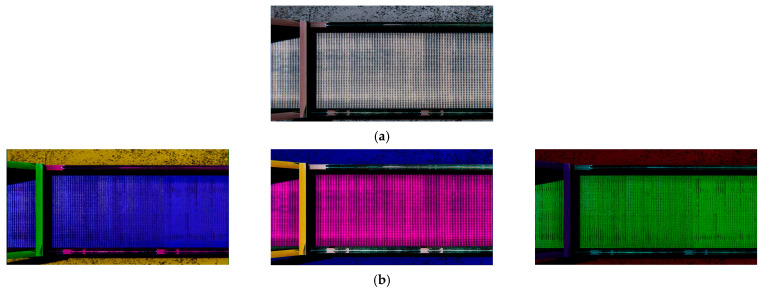
Changing the color characteristics of background objects: (**a**) conveyor line without color change; (**b**) examples of conveyor line with changed colors.

During generation, the system tracks the elapsed process time. Once the user-defined time is reached, a background object is randomly selected, and its color characteristics are modified. The color of the background object is adjusted as follows: the system sequentially modifies the values of the three RGB material color channels randomly within the range of 0.0 to 1.0. The initial background is shown in Figure 7a, while the result of modifying the background objects is shown in Figure 7b.

To simulate the operation of a conveyor line and obtain images of the object under study from various angles, it is necessary to configure the movement of the camera. The Unity game engine provides a wide range of tools for this purpose through the Cinemachine package, which is designed for image capture control and is used to automate the process of creating virtual camera movements, thereby reducing the time required to develop such solutions. The module includes implementations of algorithms for both randomly generating a motion path and tracking objects of interest. This tool significantly simplifies the process of changing viewpoints, eliminating the need for numerous manual adjustments [52].

**Figure 8:**
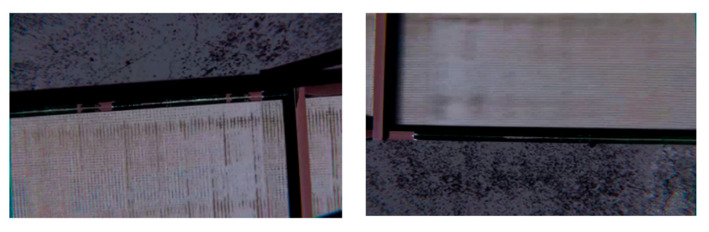
Examples of changing the camera angle.

Accordingly, as part of the synthetic data generation process, the tool described above is used to implement chaotic camera movement and capture frames from various angles, as shown in Figure 8. To achieve this, the Noise module was utilized, which enables camera movement based on a depth map generated using Perlin noise algorithms. This approach allows for the simulation of realistic motion and “shake” of the virtual camera in real time.

**Figure 9:**
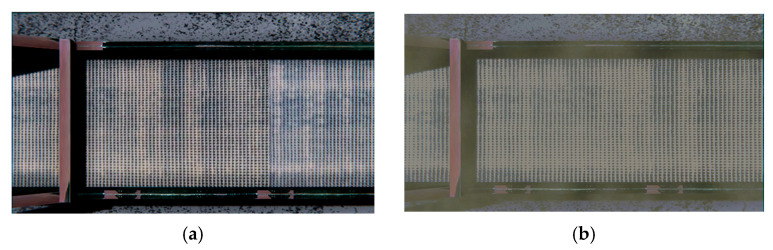
A room filled with industrial dust: (**a**) a room without dust; (**b**) a room filled with dust.

To simulate a dusty environment, the Unity Particle System module is used in the implementation. According to the game engine’s documentation, the particle system is designed to create effects from objects that do not have a defined shape and change in real time (such as smoke, fire, fluids, etc.) [53]. This description clearly applies to the “industrial dust” effect. Therefore, the particle system module is proposed for simulating dust in the environment. A dusty room with a conveyor belt is shown in Figure 9.

The developed system makes it possible to generate various production scenarios and significantly expand the final image dataset, which helps prevent issues related to model overfitting.

## 5. Testing and Results

**Figure 10:**
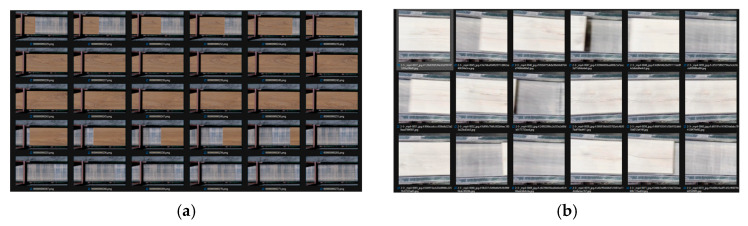
A fragment of datasets: (**a**) synthetic dataset; (**b**) real dataset.

To test the data generation method, to neural networks with the YOLOv8 architecture were trained, due to its high accuracy in object detection tasks [54]. The use of two models is driven by the need to compare a network trained exclusively on synthetic data with one trained solely on real data. Accordingly, two separate datasets were created. The synthetic dataset, a fragment of which is shown in Figure 10a, consisted of 371 generated images that were automatically annotated using the Unity Perception tool. The second dataset was created from production video recordings, split into 371 images (Figure 10b), which were manually annotated using the Roboflow tool.

Next, augmentation methods were applied to these images sets to equally expand both datasets. The final size of each dataset was 815 images. After the datasets were formed, the neural network models were treated under identical conditions in the Google Colab environment. The models were trained for 100 epochs with a batch size of 16.

To evaluate the performance of the models, we used the mean average precision (mAP) metric at a threshold of 0.5 (mAP50). This metric is composed of several components, including precision (p) and recall (r), as well as the degree of intersection over union (IoU), and average precision (AP).

Precision is the proportion of correctly identified objects among all objects that the model has detected. In other words, it is a measure of how many of the model’s predictions were correct.

(3) $$precision=\frac{TP}{TP+FP}$$

where *TP* is the number of true positive detections, and *FP* is the number of false positive detections.

*Recall* is the proportion of correctly detected objects among all objects that are actually present in the image:

(4) $$recall=\frac{TP}{TP+FN}$$

where *FN* is the number of false negative detections.

*IoU* shows how well the predicted bounding box overlaps with the real one:

(5) $$IoU=\frac{S\left(A\cap B\right)}{S\left(A\cup B\right)}$$

where *A* is the area of the predicted bounding box; *B* is the area of the true bounding box; *S* is the area of intersection or union of the bounding boxes.

The value 50 in the mAP50 metric indicates that the model’s predictions are considered correct if the *IoU* between the predicted and the ground truth bounding box is greater than 0.5 (or 50%). In other words, for a prediction to be considered successful, there must be at least 50% overlap with the actual object.

Next, a precision–recall (PR) curve was calculated for each object class, showing how precision (*p*) varied with recall (*r*). The average precision (*AP*) for each class was then computed as the area under this PR curve:

(6) $$AP=\int_{0}^{1}p\left(r\right)dr$$

As a result, after obtaining the *AP* scores for all classes, a final *mAP* score is calculated. This is the average *AP* score for all object classes:

(7) $$mAP=\frac{1}{k}\sum_{i}^{k}AP_{i}$$

where *k* is the number of classes.

As a result, if a model has an mAP50 value of 0.75, this means that on average across all object classes, detection precision (at *IoU* > 0.5) is 75%, which is considered a good result.

**Figure 11:**
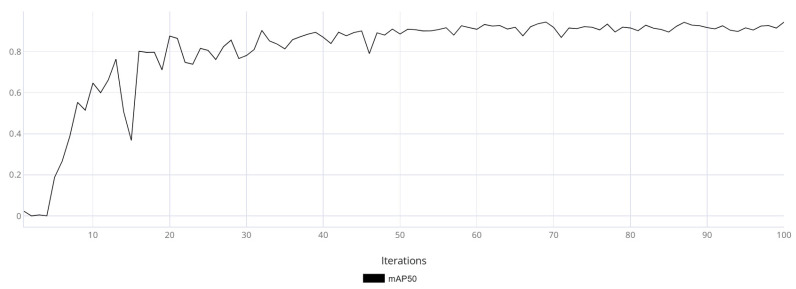
The graph of the average accuracy of classification and detection of the object of interest for a model trained on synthetic data.

**Figure 12:**
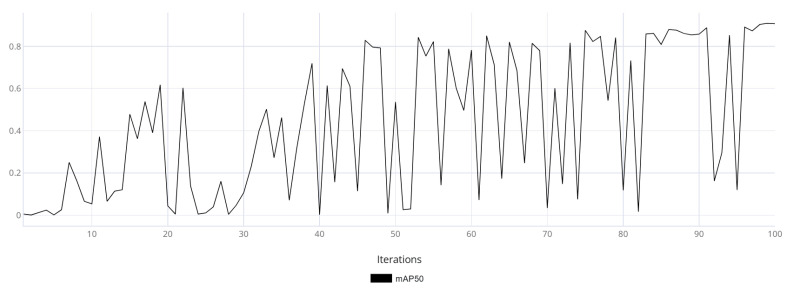
The graph of the average accuracy of the classification and detection of the object of interest for a model trained on real data.

**Figure 13:**
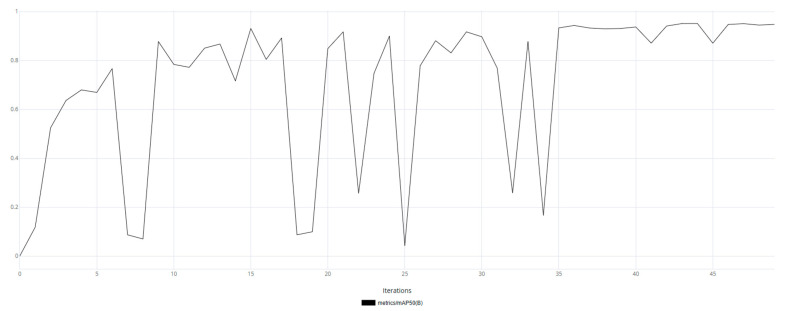
Average classification and detection accuracy for the model fine-tuned on real-world data.

The mAP50 metric variation graphs are shown for models trained on synthetic data (Figure 11) and real data (Figure 12). Additionally, training was performed on synthetic data followed by fine-tuning on real data (Figure 13).

Based on Figure 11, Figure 12 and Figure 13, it can be observed that the model trained on real data made more frequent prediction errors and reached an mAP50 of 0.93 by the end of training. While this is a good result, it does not guarantee reliability, as the uneven training curve indicated low generalization capability. In contrast, the model trained using synthetic data demonstrated a smoother training metric curve, which suggested a more robust generalization ability. The final mAP50 value for the model trained on synthetic data was 0.94. The model fine-tuned on real-world data exhibited an initial decline in accuracy; however, as training progressed, it converged toward high performance. Ultimately, the model achieved a final mAP50 score of 0.95, indicating a high level of detection precision.

After training, both models were tested on a video clip from the production environment that served as the basis for development. This video was not used to create the real data dataset, as doing so would have biased the test results.

As a result of testing, the model trained on real data achieved an mAP50 of 0.36 on the test dataset from the enterprise. This suggests that, under the same hyperparameters and image augmentation methods, the model trained on real data was not adequate. This is supported by the mAP50 training curve, which reflects the model’s inability to generalize to the presented images, indicating a lack of diversity and volume in the collected dataset. Meanwhile, the variety of the synthetic data helped overcome this issue, and the model demonstrated strong performance on the test video sequence, achieving an mAP50 of 0.95—evidence of the neural network’s adequacy. On the test video, the third model demonstrated performance comparable to that of the model trained exclusively on synthetic data. The average mAP50 on the test set reached 0.96, which was undoubtedly a strong result in terms of detecting the target object.

In conclusion, the testing results showed that the model trained on synthetic data outperformed the one trained on real data in terms of accuracy. This leads to the conclusion that the data generator is an effective tool for building computer vision systems in industrial applications.

## 6. Discussion

Based on the testing data obtained from the two models, we can draw conclusions about the performance of the synthetic data generator compared to similar solutions.

First, the proposed method for generating synthetic data stands out by providing a generalized list of tasks, significantly speeding up the development process for image generation software.

Second, compared to the methodology described in [20], our solution offers greater sampling variability thanks to the capabilities of the game engine. This allows us to recreate any desired scenarios, increasing the accuracy of models by generating images for specific tasks. In contrast to solutions that use game engines with limited capabilities like GTA V, which do not allow changing the in-game code or adding additional scenarios, our generator offers more flexible settings for modeling.

The third key aspect of the proposed system is the speed at which images are generated. The system is able to generate approximately 1500 annotated images per minute, significantly exceeding the rendering speeds of 3D modeling tools like Blender. Achieving 1 frame per second in 3D modeling is considered a good result, but the proposed solution outperforms this in terms of both speed and flexibility. In addition to the above, compared to solutions based on the Unity game engine, the proposed system demonstrates significantly better performance. Considering the speed of image generation as a single metric—the number of images generated per second—the current generator produces 25 images per second, while solutions from [24,25] produce 6 and 4.6 images per second respectively. Therefore, the performance of the proposed system is at least four times better than similar solutions.

Furthermore, it should be noted that generating synthetic data significantly speeds up the development of computer vision systems in all cases considered. This allows creating models with high accuracy using only synthetic data and further improving them by combining synthetic and real data.

At the next stage, we plan to train models to not only detect the board but also to identify and classify any defects on it. Due to the lack of test data available for the next stage during development, we have decided to focus on training models for this purpose.

## 7. Conclusions

An overview of the fields of activity where computer vision (CV) can be applied has been conducted. The analysis revealed that traditional data collection methods, such as video recording and manual image annotation, are often costly and inefficient. Moreover, these methods are subject to human error, which can lead to mistakes and reduced accuracy in neural network models. As a result, synthetic data presents an alternative that can significantly reduce the time and financial costs associated with creating training datasets.

To address the image collection task, modern approaches to generating synthetic data for automatic object detection and classification tasks on production lines using CV technologies were examined. The primary goal was to create an effective and cost-efficient solution for forming training datasets in cases of limited access to real data or insufficient data. The analysis of existing solutions showed that the use of synthetic data is becoming an increasingly relevant and sought-after tool across various industries. Various tools and technologies for generating synthetic data were reviewed, such as game engines, 3D modeling tools, and specialized packages for automatic image annotation. Each of these solutions has its advantages and disadvantages. For instance, the use of game engines allows for real-time data generation and the processing of large volumes of images at high speed, significantly accelerating the dataset creation process. On the other hand, 3D modeling provides a higher level of realism but requires significant computational resources and time for rendering.

The generalized synthetic data generator model proposed in the study was applied to implement the technological process of quality control for flooring on a conveyor line. As part of this implementation, a system was developed that simulates the real production process, taking into account all its characteristics, including camera angle changes, the addition of various defects to objects of interest, and the use of effects such as production dust. This enabled the creation of diverse and realistic images that can be used to train neural network models.

The final generation system was tested by comparing three neural network models with the YOLOv8m architecture. The use of three models was necessary to compare a network trained solely on synthetic data, one trained exclusively on real data and the model pretrained on synthetic and fine-tuned on real data. As a result, two corresponding datasets were created: the Synthetic dataset and the one based on real production video recordings.

After training the models, they were tested on a video clip from the enterprise that served as the basis for development. This video was not used to create the real data dataset, as using it would have distorted the test results. As a result of the testing, the model trained on real data achieved an mAP50 value of 0.36 on the test dataset from the enterprise. This suggests that, under the same hyperparameters and image augmentation methods, the model trained on real data was not adequate. In contrast, the diversity of synthetic data helped avoid this issue, and the model showed good performance on the test video sequence, achieving an mAP50 of 0.95, indicating the adequacy of the resulting neural network. The fine-tuning approach using real-world data demonstrated the highest performance mAP50 = 0.96, attributable to both the model’s adaptation to real-world conditions and the diversity of images present in the synthetic dataset.

Based on the testing data for the two models, conclusions can be drawn about the performance of the proposed synthetic data generator in comparison with similar solutions. Finally, it should be noted that synthetic data generation significantly accelerates the development of computer vision systems in all the reviewed cases. This allows for the creation of high-accuracy models without using real data, and further improvement through the combination of real and synthetic data.

The next phase includes research on generating artificial data for bulk materials and minerals, using coal products as an example. To solve this task, computer vision models will need to be developed and configured to train on annotated images for identifying foreign materials in piles. Real data on objects of interest (impurities in rock and foreign materials) will also need to be collected.

## Data Availability

The original contributions presented in the study are included in the article, further inquiries can be directed to the corresponding author/s.

## References

[1] Greeshma C.A., Nidhindas K.R., Sreejith P. (2019). Traffic control using computer vision. Int. J. Adv. Res. Comput. Commun. Eng..

[2] Liu G., Shi H., Kiani A., Khreishah A., Lee J., Ansari N., Liu C., Yousef M.M. (2021). Smart traffic monitoring system using computer vision and edge computing. IEEE Trans. Intell. Transp. Syst..

[3] Serrano Á., Conde C., Rodríguez-Aragón L.J., Montes R., Cabello E. Computer vision application: Real time smart traffic light. Proceedings of the Computer Aided Systems Theory–EUROCAST 2005: 10th International Conference on Computer Aided Systems Theory.

[4] Coifman B., Beymer D., McLauchlan P., Malik J. (1998). A real-time computer vision system for vehicle tracking and traffic surveillance. A real-time computer vision system for vehicle tracking and traffic surveillance. Transp. Res. Part C Emerg. Technol..

[5] Rusanovsky M., Beeri O., Oren G. (2022). An end-to-end computer vision methodology for quantitative metallography. Sci. Rep..

[6] Blackledge J., Dubovitskiy D.A. Quality Control System using Texture Analysis in Metallurgy. Proceedings of the Third International Conferences on Pervasive Patterns and Applications.

[7] Harikrishna K., Davidson M.J., Reddy G.D. (2023). New Method for Microstructure Segmentation and Automatic Grain Size Determination Using Computer Vision Technology during the Hot Deformation of an Al-Zn-Mg Powder Metallurgy Alloy. J. Mater. Eng. Perform..

[8] Sarrionandia X., Nieves J., Bravo B., Pastor-López I., Bringas P.G. (2023). An Objective Metallographic Analysis Approach Based on Advanced Image Processing Techniques. J. Manuf. Mater. Process..

[9] Aydin I., Othman N.A. A new IoT combined face detection of people by using computer vision for security application. Proceedings of the 2017 International Artificial Intelligence and Data Processing Symposium IDAP.

[10] García C.G., Meana-Llorián D., G-Bustelo B.C.P., Lovelle J.M.C., Garcia-Fernandez N. (2017). Midgar: Detection of people through computer vision in the Internet of Things scenarios to improve the security in Smart Cities, Smart Towns, and Smart Homes. Future Gener. Comput. Syst..

[11] Gupta S., Garima E. (2014). Road Accident Prevention System Using Driver’s Drowsiness Detection by Combining Eye Closure and Yawning. Int. J. Res. (IJR).

[12] Lan R., Awolusi I., Cai J. (2024). Computer Vision for Safety Management in the Steel Industry. AI.

[13] Costa C., Antonucci F., Pallottino F., Aguzzi J., Sun D.W., Menesatti P. (2011). Shape analysis of agricultural products: A review of recent research advances and potential application to computer vision. Food Bioprocess Technol..

[14] Dhanya V.G., Subeesh A., Kushwaha N.L., Vishwakarma D.K., Kumar T.N., Ritika G., Singh A.N. (2022). Deep learning based computer vision approaches for smart agricultural applications. Artif. Intell. Agric..

[15] Arakeri M.P. (2016). Computer vision based fruit grading system for quality evaluation of tomato in agriculture industry. Procedia Comput. Sci..

[16] Esteva A., Chou K., Yeung S., Naik N., Madani A., Mottaghi A., Liu Y., Topol E., Dean J., Socher R. (2021). Deep learning-enabled medical computer vision. NPJ Digit. Med..

[17] Villalba-Diez J., Schmidt D., Gevers R., Ordieres-Meré J., Buchwitz M., Wellbrock W. (2019). Deep learning for industrial computer vision quality control in the printing industry 4.0. Sensors.

[18] Conrad J., Rodriguez S., Omidvarkarjan D., Ferchow J., Meboldt M. (2023). Recognition of Additive Manufacturing Parts Based on Neural Networks and Synthetic Training Data: A Generalized End-to-End Workflow. Appl. Sci..

[19] de Melo C.M., Torralba A., Guibas L., DiCarlo J., Chellappa R., Hodgins J. (2022). Next-generation deep learning based on simulators and synthetic data. Trends Cogn. Sci..

[20] Kiefer B., Ott D., Zell A. Leveraging synthetic data in object detection on unmanned aerial vehicles. Proceedings of the 2022 26th International Conference on Pattern Recognition (ICPR).

[21] Blender Foundation Blender—Free and Open Source 3D Creation Software. https://www.blender.org/.

[22] Maxon ZBrush—Digital Sculpting & Painting Software. https://www.maxon.net/en/zbrush.

[23] Autodesk 3ds Max—3D Modeling, Animation & Rendering Software. https://www.autodesk.com/products/3ds-max/overview.

[24] Chumak R. A Synthetic Data Generator. Training of Neural Networks for Industrial Flaw Detection. https://medium.com/phygitalism/synthetic-data-generator-a052d347468.

[25] Schmedemann O., Baaß M., Schoepflin D., Schüppstuhl T. (2022). Procedural synthetic training data generation for AI-based defect detection in industrial surface inspection. Procedia CIRP.

[26] Reutov I., Moskvin D., Voronova A., Venediktov M. (2022). Generating Synthetic Data To Solve Industrial Control Problems by Modeling A Belt Conveyor. Procedia Comput. Sci..

[27] Lee H., Jeon J., Lee D., Park C., Kim J., Lee D. (2023). Game engine-driven synthetic data generation for computer vision-based safety monitoring of construction workers. Autom. Constr..

[28] Pchelintsev S., Yulyashkov M.A., Kovaleva O.A. (2022). A method for creating synthetic datasets for training neural network models to recognize objects. Inf. Manag. Syst..

[29] Manyar O.M., Cheng J., Levine R., Krishnan V., Barbič J., Gupta S.K. (2023). Physics Informed Synthetic Image Generation for Deep Learning-Based Detection of Wrinkles and Folds. ASME J. Comput. Inf. Sci. Eng..

[30] Varol G., Romero J., Martin X., Mahmood N., Black M.J., Laptev I., Schmid C. Learning from synthetic humans. Proceedings of the IEEE Conference on Computer Vision and Pattern Recognition (CVPR).

[31] Hinterstoisser S., Lepetit V., Wohlhart P., Konolige K. On pre-trained image features and synthetic images for deep learning. Proceedings of the European Conference on Computer Vision (ECCV) Workshops.

[32] Hou X., Sun K., Shen L., Qiu G. (2024). Feature Perceptual Loss for Variational Autoencoder. https://arxiv.org/pdf/1610.00291.

[33] Creswell A., White T., Dumoulin V., Arulkumaran K., Sengupta B., Bharath A. (2017). Generative Adversarial Networks: An Overview. https://arxiv.org/pdf/1710.07035.

[34] Tripathi S., Chandra S., Agrawal A., Tyagi A., Rehg J.M., Chari V. Learning to generate synthetic data via compositing. Proceedings of the IEEE/CVF Conference on Computer Vision and Pattern Recognition.

[35] Saiz F.A., Alfaro G., Barandiaran I., Graña M. (2021). Generative Adversarial Networks to Improve the Robustness of Visual Defect Segmentation by Semantic Networks in Manufacturing Components. Appl. Sci..

[36] Goncharov A.S., Goncharov A.S., Saklakov V.M. (2018). Digital twin: An overview of existing solutions and prospects for technology development. Information and Telecommunication Systems and Technologies, Proceedings of the All-Russian Scientific and Practical Conference, Kemerovo, 11–13 October 2018.

[37] Kritzinger W., Karner M., Traar G., Henjes J., Sihn W. (2018). Digital Twin in manufacturing: A categorical literature review and classification. IFAC-PapersOnLine.

[38] Koroteev D.D., Kim A.A., Vasyutin A.O. (2024). Prospects for the use of digital twins in the construction industry. Eurasian Sci. J..

[39] Vikhman V.V., Romm M.V. (2021). “Digital twins” in education: Prospects and reality. High. Educ. Russ..

[40] Verdouw C., Tekinerdogan B., Beulens A., Wolfert S. (2021). Digital twins in smart farming. Agric. Syst..

[41] Bhatti G., Mohan H., Singh R.R. (2021). Towards the future of smart electric vehicles: Digital twin technology. Renew. Sustain. Energy Rev..

[42] Erdélyi V., Jánosi L. (2019). Digital Twin and Shadow in Smart Pork Fetteners. Int. J. Eng. Manag. Sci..

[43] Case Study: VDL Nedcar, Perspective Software. https://prespective-software.com/case-studies/case-study-vdl-nedcar/.

[44] Huang Y., Yuan B., Xu S., Han T. (2022). Fault Diagnosis of Permanent Magnet Synchronous Motor of Coal Mine Belt Conveyor Based on Digital Twin and ISSA-RF. Processes.

[45] Singh M., Kapukotuwa J., Gouveia E.L.S., Fuenmayor E., Qiao Y., Murray N., Devine D. (2025). Comparative Study of Digital Twin Developed in Unity and Gazebo. Electronics.

[46] Pujana A., Esteras M., Perea E., Maqueda E., Calvez P. (2023). Hybrid-Model-Based Digital Twin of the Drivetrain of a Wind Turbine and Its Application for Failure Synthetic Data Generation. Energies.

[47] Digital Twin: Applications and Use Cases, Unity. https://unity.com/ru/topics/digital-twin-applications-and-use-cases.

[48] Dustler M., Bakic P., Petersson H., Timberg P., Tingberg A., Zackrisson S. Application of the fractal Perlin noise algorithm for the generation of simulated breast tissue. Proceedings of the Medical Imaging 2015: Physics of Medical Imaging.

[49] Bazuhair W., Lee W. Detecting malign encrypted network traffic using perlin noise and convolutional neural network. Proceedings of the 2020 10th Annual Computing and Communication Workshop and Conference (CCWC).

[50] Li H., Tuo X., Liu Y., Jiang X. A parallel algorithm using Perlin noise superposition method for terrain generation based on CUDA architecture. Proceedings of the International Conference on Materials Engineering and Information Technology Applications (MEITA 2015).

[51] Ying X. (2019). An overview of overfitting and its solutions. J. Phys. Conf. Ser..

[52] About Cinemachine. https://docs.unity3d.com/Packages/com.unity.cinemachine@2.8/manual/index.html.

[53] Particle System. https://docs.unity3d.com/ru/530/Manual/ParticleSystems.html.

[54] Sohan M., Sai Ram T., Reddy R., Venkata C. A review on yolov8 and its advancements. Proceedings of the International Conference on Data Intelligence and Cognitive Informatics.

